# Effects of seated lumbar rotation manipulation in treating degenerative lumbar instability: a protocol for a randomized controlled trial

**DOI:** 10.1186/s13063-021-05350-1

**Published:** 2021-06-14

**Authors:** Rui Xie, Long Liang, Kaiming Li, Jie Yu, Minshan Feng, Jiawen Zhan, Xu Wei, Kexin Yang, Zhefeng Jin, He Yin, Xin Chen, Xunlu Yin, Zhiwei Liu, Wenkang Dai, Liguo Zhu

**Affiliations:** 1 Department of Spine, Wangjing Hospital of China Academy of Chinese Medical Sciences, Beijing, 100102 China; 2 Beijing Key Laboratory of Orthopedics of Traditional Chinese Medicine, Wangjing Hospital of China Academy of Chinese Medical Sciences, Beijing, China; 3 Department of Rehabilitation, Wangjing Hospital of China Academy of Chinese Medical Sciences, Beijing, China; 4 Office of Academic Research, Wangjing Hospital of China Academy of Chinese Medical Sciences, Beijing, China

**Keywords:** Degenerative lumbar instability, DLI, Seated lumbar rotation manipulation, Protocol, Clinical trial, Orthopaedic, Spine, Musculoskeletal disorders

## Abstract

**Background:**

Degenerative lumbar instability (DLI) is a common disease that causes low back pain (LBP) in clinic. It is difficult to completely recover from DLI, and it occurs repeatedly, which seriously affects the quality of life of patients. The epidemiological survey showed that 20–30% of low back pain was related to lumbar instability. Increasing evidence shows that seated lumbar rotation manipulation can effectively improve the clinical symptoms of patients with low back pain. The primary aim of this clinical trial is to observe the intervention effect of seated lumbar rotation manipulation on DLI patients.

**Method/design:**

A total of 60 participants with DLI will be recruited and randomly allocated into the seated lumbar rotation manipulation group (the intervention group) or lumbar traction in supine position group (the control group) in this prospective, outcome assessor-blind, two-arm randomized controlled clinical trial. The treatment of the two groups lasted for 3 weeks, and the manipulation of the intervention group would be carried out once every other day, three times a week, a total of 9 times; the control group would be given lumbar traction once a day, five times a week, a total of 15 times. JOA (Japanese Orthopaedic Association) and VAS (Visual Analogue Scales) scores will be recorded as the primary outcomes before the treatment and at the 1st, 3rd, 5th, 8th, 10th, 12th, 15th, 17th, and 19th days after treatment and follow-up visit at the first, third, and sixth months. JOA efficacy evaluation standard will be used to evaluate the overall efficacy as the secondary outcomes.

**Discussion:**

The results of this prospective, randomized controlled trial will provide a clinical evidence for the treatment of DLI with seated lumbar rotation manipulation.

**Trial registration:**

Chinese Clinical Trial Registry ChiCTR2000032017. Registered on 18 April 2020, Prospective registration.

## Introduction

Degenerative lumbar instability (DLI) is a common disease that causes low back pain in clinic. It is difficult to completely recover from DLI, and it occurs repeatedly, which seriously affects the quality of life of patients. According to the data of the World Health Organization global disease burden research [1], among 301 diseases in 188 countries, low back pain ranks first in the disease burden indicators of developed and underdeveloped countries all the year round. In China, the disease burden caused by low back pain also ranks first. The epidemiological survey [2] shows that 20–30% of low back pain is related to lumbar instability, which shows that the disease brings huge economic and social burden.

The treatment of DLI includes non-surgical (conservative) treatment and surgical treatment. The choice of the two mainly depends on the degree of lumbar instability, the type of involved tissue, secondary deformity, the risk of complications, and psychological, social, economic and other comprehensive factors. Clinical conservative treatment of most patients with DLI can achieve good results [3, 4]. Absolutely lying in bed or strengthening the functional exercises of lumbar, back, and abdominal muscles can achieve the purpose of prevention and treatment of back pain. However, surgical treatment is limited to patients with obvious neurological symptoms, progressive aggravation of spondylolisthesis, and ineffective conservative treatment. It is generally believed that non-surgical treatment is the first choice for the treatment of DLI, such as bed rest, brace, manipulation, traction, physical therapy, and needle-knife.

The traditional Chinese manipulation has unique advantages in the treatment of lumbar and back pain, and the clinical reports [5–8] on the treatment of low back pain, lumbar disc herniation, lumbar facet joint disorder, lumbar spondylolisthesis, and other diseases with sitting rotation manipulation also show the effectiveness of this method.

Combined with the mechanism research of the manipulation [9, 10], the author believes that in the clinical treatment, first of all, the muscle can be relaxed directly through the doctor’s massage, which can achieve the purpose of loosening the adherent tissue and relieving the spasm. This can promote blood circulation and improve the ischemic and anoxic state of the lesion. At the same time, the increase of local tissue temperature can promote the repair of injured tissue and then promote the absorption of hematoma and edema caused by injury. In the application of manipulation, by rotating and pulling force, the facet joint produces prying movement, increases the intervertebral foramen, relieves the compression on the nerve root, adjusts the stress distribution of the spinal movement segment, and reconstructs the mechanical balance. In the stable position, the spine regained its stability through self-repair.

In order to evaluate the clinical effect of seated lumbar rotation manipulation in the treatment of DLI, we plan to design a prospective, randomized controlled trial. The main purpose of this experiment is to determine the influence of seated lumbar rotation manipulation on the activity limitation and clinical signs of patients with DLI.

Supine lumbar traction is one of the common treatment methods in the clinical treatment of DLI. The main function of traction is to separate the articular surface and traction the surrounding soft tissue, so as to expand the lumbar vertebral space, change the bone structure, reduce the pressure in the spinal canal, relieve the compression state of the spinal cord and peripheral nerve, and achieve the purpose of reducing the symptoms of patients. In most of the clinical studies of manipulation in the treatment of degenerative lumbar diseases, supine lumbar traction was used as the control group [11, 12].

We hypothesized that compared with the intervention of lumbar traction in supine position, seated lumbar rotation manipulation would improve the symptoms, physical signs, and activity limitation of patients.

## Method/design

### Study design

According to the random number table method [13], a total of 60 patients with DLI will be divided into sitting lumbar rotation manipulation group (the intervention group) and supine lumbar traction group (the control group), 30 cases in each group. DLI patients will be numbered according to the visit time, and any one number in the random number table will be selected as the starting point. A random number corresponding to each patient will be obtained along the same direction. Divide this number by 2, and the remainder of 0 will be the intervention group, and the remainder of 1 will be the control group. Two residents participating in this study will be responsible for generating the allocation order sequence.

**Fig. 1:**
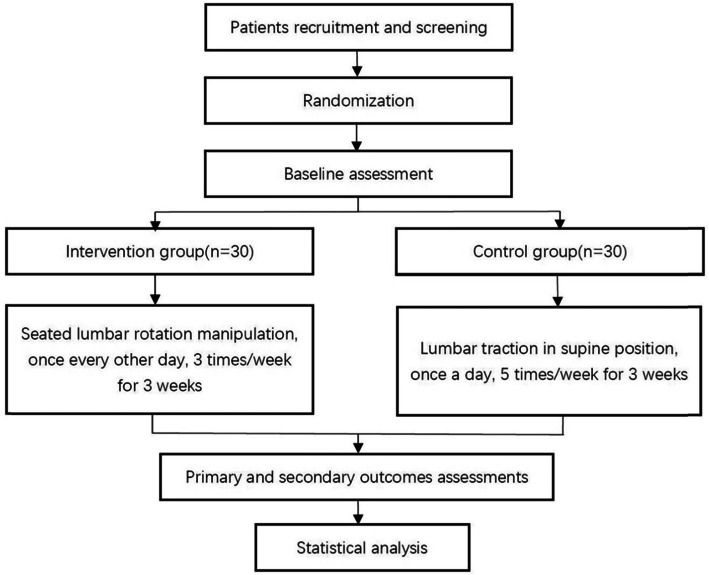
Flow chart of the trial

**Fig. 2:**
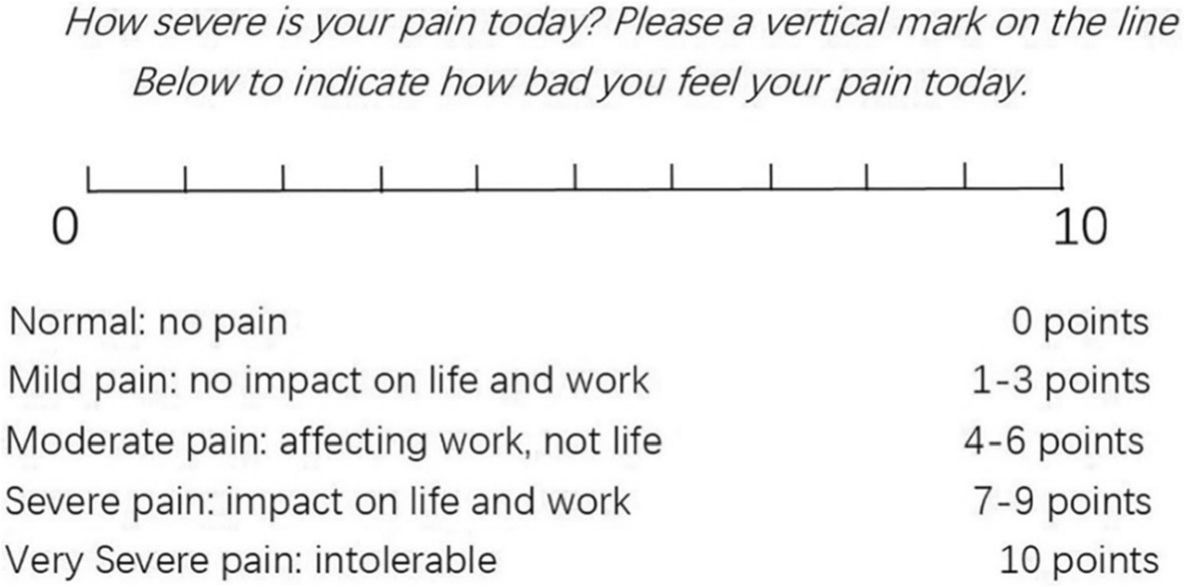
visual analogue scales(VAS) [15]

The treatment of the two groups lasted for 3 weeks. The manipulation of the intervention group would be carried out once every other day, three times a week, a total of 9 times; the control group would be given lumbar traction once a day, five times a week, a total of 15 times. JOA [14] and VAS [15] scores will be recorded as the primary outcomes before the treatment and at the 1st, 3rd, 5th, 8th, 10th, 12th, 15th, 17th, and 19th days after the first treatment and follow-up visit at the 1st, 3rd, and 6th months. JOA [14] efficacy evaluation standard will be used to evaluate the overall efficacy as the secondary outcomes. When analyzing the results, the personnel who collect the data are blind to the grouping situation. The flow of this clinical trial is shown in Fig. 1; the schedule of enrolment, assessments, and interventions is presented in Table 1. JOA [14] and VAS [15] scores are presented in Table 2 and Fig. 2.

**Table 1:**
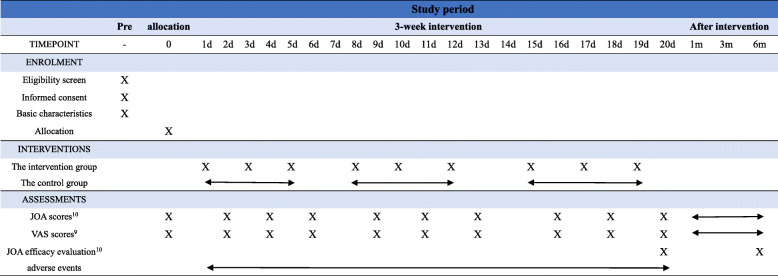
Schedule of enrolment, interventions, and assessments

**Table 2:** The JOA [14] scoring system for assessment of low back pain. The score in a normal subject is the total of the best scores: (I+II+III+IV) = 29 points

Parameter	Finding	Score (points)
I. Subjective symptoms (9 points)		
a. Low back pain	None	3
	Occasional mild pain	2
	Frequent mild/occasional severe pain	1
	Frequent pain/continuous pain	0
b. Leg pain and/or tingling	None	3
	Occasional slight symptoms	2
	Frequent slight symptoms/occasional severe symptoms	1
	Frequent severe symptoms/continuous severe symptoms	0
c. Gait	Normal	3
	Able to walk > 500 meters although it results in pain tingling and/or muscle weakness	2
	Unable to walk > 500 meters owing to leg pain tingling and/or muscle weakness	1
	Unable to walk > 100 meters owing to leg pain tingling and/or muscle weakness	0
II. Clinical signs (6 points)		
a. Straight leg raising (includes a tight hamstring)	Normal (> 70°)	2
	30° to 70°	1
	< 30°	0
b. Sensory disturbance	None	2
	Slight disturbance (not subjective)	1
	Marked disturbance	0
c. Motor disturbance	Normal (Grade 5)	2
	Slight weakness (Grade 4)	1
	Marked weakness (Grades 0 to 3)	0
III. Restriction in activities (14 points)		
a. Turn over while lying	No restriction	2
	Moderate restriction	1
	Severe restriction	0
b. Standing	No restriction	2
	Moderate restriction	1
	Severe restriction	0
c. Washing	No restriction	2
	Moderate restriction	1
	Severe restriction	0
d. Leaning forward	No restriction	2
	Moderate restriction	1
	Severe restriction	0
e. Sitting about 1 h	No restriction	2
	Moderate restriction	1
	Severe restriction	0
f. Lifting or holding a heavy object	No restriction	2
	Moderate restriction	1
	Severe restriction	0
g. Walking	No restriction	2
	Moderate restriction	1
	Severe restriction	0
IV. Urinary bladder function (−6 points maximum)	Normal	0
	Mild dysuria	-3
	Severe dysuria	-6

### Study population

The study population is the patients admitted to the orthopaedic ward and orthopaedic clinic of Wangjing Hospital, Chinese Academy of Traditional Chinese Medicine. The diagnostic, inclusion, exclusion, rejection, and termination criteria for this trial sample are as follows.

#### Diagnostic criteria

According to Kirkaldy-Willis’ statement of spinal instability [16], combined with the reports of many scholars [17–19], the clinical diagnostic criteria of DLI are as follows:(1) obvious and repeated low back pain, and severe pain or weakness; (2) local low back pain and/or pain associated with lower extremity involvement; (3) MRI, CT, and other examinations show obvious degeneration of lumbar disc and joint, which exclude other diseases; and (4) X-ray: (1) the leading edge of vertebral body showed distraction osteophyte formation or obvious stenosis of intervertebral space and (2) in the dynamic radiograph (hyperextension, hyperflexion lateral radiograph) of the lumbar spine, the slip between the adjacent two vertebrae was more than 3 mm (but the forward or backward slip of the upper vertebrae was not more than 1/4 of the sagittal diameter of the middle vertebrae, and the lesion was not more than 2 segments), or the angle between the adjacent vertebrae was more than 11°.

#### Inclusion criteria

(1) Diagnosed with DLI, (2) aged from 45 to 65 years old, and (3) provided informed consent.

#### Exclusion criteria

(1) Instability caused by congenital spinal diseases; (2) instability caused by spinal infection, traumatic fracture, tumor, tuberculosis, and osteoporosis; (3) with serious heart, cerebrovascular, hepatorenal and hematopoietic system and endocrine system diseases; (4) patients with mental illness or Alzheimer’s disease; (5) lumbar instability of dysplasia, isthmus, trauma, and pathology; and (6) severe skin injury or skin disease at the manipulation site

#### Rejection criteria

(1) The patients do not cooperate with the randomized program and do not receive treatment after randomization; (2) patients do not meet the inclusion criteria and/or exclusion criteria; (3) there is no treatment or any record of patients after enrollment; and (4) during the observation period, the patients received treatment by methods other than the protocol.

#### Termination criteria

(1) Serious adverse events (life-threatening or affecting normal general work and life) occur; (2) in the course of treatment, the symptoms disappear completely and the patients are unwilling to receive treatment again; and (3) patients with severe and persistent anaphylaxis will stop treatment and be treated as invalid cases.

### Recruitment

Participants will be recruited by the spine doctors of Wangjing Hospital of the Chinese Academy of Traditional Chinese Medicine through posters, design of network recruitment, and sending flyers. According to the inclusion and exclusion criteria mentioned above, 2 chief physicians in this clinical trial will select appropriate patients to join the study.

There will be trained staff to discuss the informed consent with the enrolled patients.

After obtaining consent and signing informed consent form, baseline assessment shall be conducted.

### Randomization, allocation concealment, and blinding

Upon completion of the baseline assessment, participants were randomly assigned by the physician to the sitting lumbar rotation manipulation group and the supine lumbar traction control group. Because doctors and patients are able to know which kind of intervention will be accepted, they cannot be blind to the treatment, but the result evaluator and data statistician are blind to the grouping and treatment.

### Intervention

#### Seated lumbar rotation manipulation group

The seated/sitting lumbar rotation manipulation [20] treatment in this group will be divided into three parts, which are examination, preparation and treatment technique. It will be carried out once every other day, three times a week, a total of 9 times.

- Examination technique

Patients stand straight, feet apart and shoulder width, hands naturally droop, exposing the waist and back. The doctor observed whether there was physiological curvature of the spine, whether there was scoliosis, and then palpated with index finger, middle finger, and ring finger. Doctors use the middle finger to move from top to bottom along the apex of the spinous process. The index finger and ring finger are on both sides of the middle finger. They can feel whether there is any abnormality in the arrangement of spinous processes, whether there is a deviation of spinous processes to one side, whether the adjacent spinous processes are not in a straight line, or step sense. Make the patient lumbar flexion and extension, and then carry out the above examination. Doctors combined with imaging examination and physical examination to determine the abnormal stage of lumbar spine and the oblique position of spinous process.

b. Preparation technique

The patient is in a prone position and relaxed. The doctor’s hands are placed on both sides of the patient’s spine, on the surface of the sacrospinalis muscle, from the outside to the inside, from the shallow to the deep, pushing and rubbing the vertical spine muscle rhythmically, and shaking the lumbar spine left and right at the same time. The function of this method is to relax muscles, relieve muscle spasm, release adhesion, activate blood circulation and remove blood stasis, dredge channels and collaterals, and relieve spasm and pain. Take the next step when your muscles are relaxed.

iii. Treatment technique

**Fig. 3:**
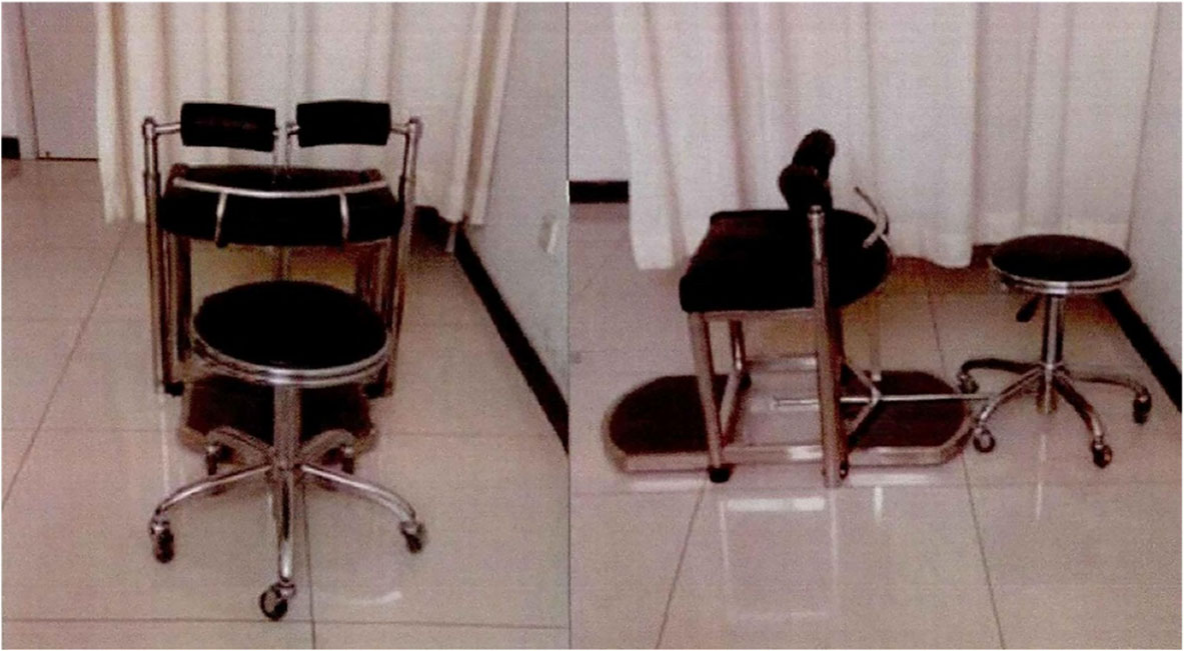
Controllable treatment chair

**Fig. 4:**
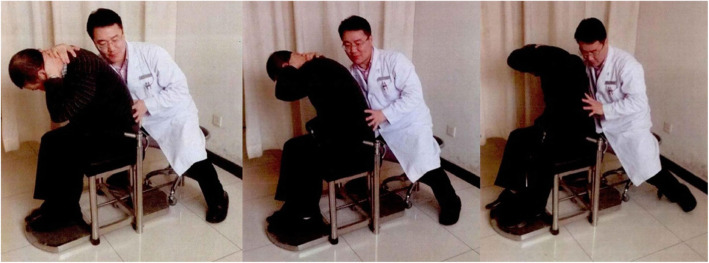
Seated lumbar rotation manipulation (flexion, rotation, and pulling)

The doctor will use the controllable treatment chair (Fig. 3) for manual treatment. According to the height and weight of the patient and the height of the doctor, the height and distance of the patient chair and the doctor chair are adjusted by using the calculation results of the parameter formula. The patient sits on this special treatment chair, relaxes the waist with both legs fixed. The doctor puts one hand against the spinous process of the unstable segment of the lumbar spine, and the other hand passes through the armpit of one side of the patient, pressing the neck and shoulder of the other side. First, let the patient slowly bend the spine forward. When the doctor’s thumb feels the interspace of spinous process open, maintain this range. Then let the patient rotate to this side as much as possible. Finally, the doctor flexes and rotates the patient’s lumbar vertebrae with the hand pressed on the neck and shoulder. The thumb of the other hand pushes the spinous process of the abnormal vertebral body. At this time, it can often be heard the “click” sound, and the doctor can feel the spike jump under the thumb holding the spike. Repeat the same maneuver on the opposite side. The treatment technique can be simply summarized into three parts: flexion, rotation, and pulling, as shown in Fig. 4.

The manipulators are trained doctors. They will receive a 2-week training, and the clinical research can only be carried out after they pass the examination of the chief physician.

#### Lumbar traction in supine position control group

The operation of the lumbar traction in supine position [21] treatment method is as follows: the patient is in supine position with the chest band fixed in both armpits and the pelvic band fixed in the upper edge of the iliac crest. The tightness of binding is moderate. The traction weight starts from a small amount and increases to 50% of the patient’s weight. During traction, a triangular brace is placed in the lumbosacral part of the patient, which is located at the lower edge of the lumbar sliding instability segment. It will be taken once a day, 5 times a week, 30 min each time.

Implementing seated lumbar rotation manipulation and lumbar traction in the supine position group will not require alteration to usual care pathways (including use of any medication) and these will continue for both trial arms.

### Outcome assessment

Variables for this clinical trial included the patient’s baseline, primary, and secondary outcomes.

The baseline will be completed before treatment, and the primary and secondary results will be measured at baseline and on the 1st, 3rd, 5th, 8th, 10th, 12th, 15th, 17th, and 19th days after the first treatment, and followed up at the 1st, 3rd, and 6th months. All primary and secondary outcomes will be evaluated by experienced doctors at Wangjing Hospital, and patient allocation results will be blind to them.

#### Basic information

Researchers will use a self-designed questionnaire to collect participants’ demographic characteristics (such as gender, age, education, marital status, height, and weight) and medical history.

#### Primary outcomes

We will use the Japanese Department of orthopedics Association (JOA [14]) low back pain rating scale (Table 1) to observe and record the symptom and sign scores of the two groups of patients at each visit point. The parameters in the scoring table are divided into four parts. The maximum score is 29 points, and the minimum score is −6 points. The higher the score the more normal the patient’s overall status. VAS [15] will be used to observe and record the pain degree of each visiting point in the two groups (Fig. 2), which means marking the numbers 0–10 with a straight line divided into 10 equal parts, according to the patient’s degree of pain to determine and draw on the corresponding number, and finally by the doctor according to the patient’s mark on the scale of the number to score. If the patient is marked on 5, it will be recorded as 5 points.

#### Secondary outcomes

JOA [14] efficacy evaluation standard will be used to evaluate the overall efficacy as the secondary outcomes, which is the calculation of treatment improvement rate. Treatment improvement percentage= (post-treatment score) − (pre-treatment score)/(29 −(pre-treatment score)) × 100%. When the improvement rate is 100%, means it is cured; more than 60%, effective; 25–60%, effective; and less than 25%, invalid.

### Safety measurements

During the intervention period, researchers will monitor the occurrence of adverse events; observe the type, degree, and incidence of adverse events; and record and handle them. If there are any adverse events, such as trauma, the adverse event case report form (CRF) will be used for detailed reporting. In addition, the researchers will specifically note the causal relationship and severity of adverse events in the evaluation of seated lumbar rotation manipulation intervention. If serious adverse events occur, they will be reported to the ethics committee.

### Sample size

As there is no literature reference to calculate the sample size of DLI treated by sitting lumbar rotation manipulation, and few clinical and statistical assumptions supporting the sample size calculations, so this study is an exploratory study. No references for the experience accumulation method indicated, so we selected 60 cases of small sample.

### Statistical analysis

An independent statistician will use the SPSS V.25.0 software to perform the statistical analysis. Count data and grade data will be described by the number of case. The counting data will be tested by χ^2^; Wilcoxon rank-sum test will be applied to rank data. The measurement data will be statistically described with mean ± standard deviation. In group comparison, paired sample t-test will be applied. The rank sum test will be used for the difference comparison between groups. *P* < 0.05 will be considered statistically significant.

### Data collection and management

The outcome assessors will use the CRF papers to collect the data. The CRF papers will be randomly put into envelopes of different groups and sent to a researcher who is responsible for electronic data input. Then, the researcher will transcribe the CRF into an electronic data acquisition system (EDC), which is a free Research Manager named ResMan (http://www.medresman.org) provided by the China Clinical Trial Registry and meets the available standards for security. Every subject will be assigned a separate experiment identification number, and the relevant data will be strictly confidential. Only members of the trial will have access to the data set. Anonymized trial data will be shared with other researchers to enable international prospective meta-analyses. The research data will be stored in the EDC system in a separate password-protected location. Data Monitoring Committee was not considered as this is a low-risk intervention.

## Discussion

Instability caused by lumbar degeneration is often seen in clinical diagnosis and treatment. Due to the narrowing of the intervertebral space, the lateral recess is relatively narrow. At the same time, the degeneration can cause the movement fulcrum of lumbar vertebrae to move back to the facet joint, making the facet joint degenerate and thicken, the facet joint subluxate, resulting in the narrowing of the lateral recess and spinal canal, the corresponding narrowing of the nerve root outlet, and stimulating the nerve root to produce back pain and other symptoms. These changes are aggravated during exercise, and symptoms such as low back pain and leg pain are aggravated when nerve roots are stimulated.

In order to ensure the effective and complete data, but also to protect the interests of the subjects, we will improve the compliance of the subjects from the following aspects. (a) When the subjects were screened, more telephone numbers of their family members were left to facilitate communication. During the experiment, all the questions will be answered in time, and the information will be collected and reported to the researchers. (b) Make the appointment of project examination, the preparation of application form and the record of data in advance to ensure that the subjects can enter the treatment course in time. Patiently explain the possible complications in the treatment, and tell the subjects not to be nervous once this happens, and the doctor will take necessary measures. (c) Humanistic care. During the manipulation, patients’ nervous mood will be appeased, and appropriate psychological comfort will be given, especially for older subjects. When it is time for treatment, telephone notice or SMS greetings will be given in advance. During the treatment, the patient’s condition will be continuously observed and reported to the researcher when necessary. At the same time, we will promote participant retention and compete follow-up in the above ways.

If the subjects could not complete the whole process of the trial due to any reasons, the reasons for their withdrawal should be recorded in detail. At the same time, attention should be paid to these patients for further observation and treatment within a certain period of time to protect the safety of patients after withdrawal from the trial. For the missing data processing, multiple imputation (MI) method [22] will be used to complete the missing data.

However, it takes time and experience for young doctors to master this method. The inheritance of manual operation is mostly carried out by oral and psychological teaching, which lacks the support of objective technical indicators. It takes a lot of time for beginners to practice and explore in clinical practice, so the learning efficiency is low, and the effect could not be evaluated by standards. In this way, the whole manipulation process is quantified through mechanical analysis, making it an objective evaluation method. This is of great significance to the experience inheritance, teaching training, and application promotion of clinical manipulation [23–27]. The mechanical analysis and kinematic analysis of the sitting lumbar rotation manipulation show that the motion capture technology can measure, capture, and record the movement track of the object in the three-dimensional space, which makes the three-dimensional dynamic analysis tend to high precision and visualization [28, 29]. In an experiment of using this manipulation to treat degenerative lumbar spondylolisthesis [9], it was found that there was no significant difference in the mechanical parameters of the left and right hands of the operator, so it was considered that there was no significant difference in the manipulation of the left and right hands in clinical practice. Through multiple linear regression analysis, it is found that age is the most obvious factor affecting the manipulation time and height is the most obvious factor influencing the maximum speed and acceleration. Therefore, the manual operator should adjust the hair force according to the patients of different ages and body shapes to implement different forces. For example, the manual operation should be gentle for the elderly and the patients with relatively small body shapes, and the strength and speed should be increased for the young and tall patients.

## Trial status

The protocol version number is Version 1.0, 30 April 2020. The recruitment began on 1 May 2020; the expected date for recruitment completion is 1 May 2021.

## Data Availability

No additional data available.

## Abbreviations

DLI: Degenerative lumbar instability
SLRM: Seated lumbar rotation manipulation
JOA: Japanese Department of orthopedics Association
VAS: Visual Analogue Scales/scores
SPIRIT: Standard Protocol Items: Recommendations for Interventional Trials
TCM: Traditional Chinese medicine
